# Complete Characterization of the O-Antigen from the LPS of *Aeromonas bivalvium*

**DOI:** 10.3390/ijms23031204

**Published:** 2022-01-21

**Authors:** Rossella Di Guida, Angela Casillo, Juan M. Tomás, Susana Merino, Maria Michela Corsaro

**Affiliations:** 1 Department of Chemical Sciences, University of Naples “Federico II”, Complesso Universitario Monte S. Angelo, Via Cintia 4, 80126 Naples, Italy; ross.diguida@gmail.com (R.D.G.); angela.casillo@unina.it (A.C.); 2 Departamento de Genética, Microbiología y Estadística, Sección Microbiología, Virología y Biotecnología, Facultad de Biología, Universidad de Barcelona, Avd. Diagonal 643, 08028 Barcelona, Spain; jtomas@ub.edu (J.M.T.); smerino@ub.edu (S.M.)

**Keywords:** *Aeromonas*, lipopolysaccharide, O-antigen, structure elucidation, NMR spectroscopy

## Abstract

*Aeromonas* species are found in the aquatic environment, drinking water, bottled mineral water, and different types of foods, such as meat, fish, seafood, or vegetables. Some of these species are primary or opportunistic pathogens for invertebrates and vertebrates, including humans. Among the pathogenic factors associated with these species, there are the lipopolysaccharides (LPSs). LPSs are the major components of the external leaflet of Gram-negative bacterial outer membrane. LPS is a glycoconjugate, generally composed of three portions: lipid A, core oligosaccharide, and O-specific polysaccharide or O-antigen. The latter, which may be present (smooth LPS) or not (rough LPS), is the most exposed part of the LPS and is involved in the pathogenicity by protecting infecting bacteria from serum complement killing and phagocytosis. The O-antigen is a polymer of repeating oligosaccharide units with high structural variability, particularly the terminal sugar, that confers the immunological specificity to the O-antigen. In this study, we established the structure of the O-chain repeating unit of the LPS from *Aeromonas bivalvium* strain 868 E^T^ (=CECT 7113^T^ = LMG 23376^T^), a mesophilic bacterium isolated from cockles (*Cardium* sp.) and obtained from a retail market in Barcelona (Spain), whose biosynthesis core LPS cluster does not contain the *waaE* gene as most of *Aeromonas* species. After mild acid hydrolysis, the lipid A was removed by centrifugation and the obtained polysaccharide was fully characterized by chemical analysis and NMR spectroscopy. The polymer consists of a heptasaccharide repeating unit containing D-GalNAc, L-Rha, D-GlcNAc, and D-FucNAc residues.

## 1. Introduction

The genus *Aeromonas* belongs to the *Aeromonadaceae* family and comprises Gram-negative bacteria widely distributed in aquatic environments [1]. They are rod-shaped and facultative anaerobic bacteria with an optimal growth temperature range between 22 °C and 37 °C. Based on the latter parameter, *Aeromonas* spp. were divided into two major groups: mesophiles and psychrophiles [2].

They are considered emerging pathogens especially in mammals and fish. In humans, they cause a variety of extraintestinal and systemic infections, as gastrointestinal infections. Most of the clinical isolates of the genus *Aeromonas* include the four following mesophilic species: *A. hydrophila*, *A. caviae*, *A. dhakensis*, and *A. veronii* [2]. Their pathogenesis depends on a wide range of virulence factors, such as the type III secretion system, polar and lateral flagella, and the surface components of the outer membrane (i.e., the S-layer, the capsular polysaccharide, and the lipopolysaccharide) [3,4].

Lipopolysaccharide (LPS) is the major component of the external leaflet of the bacterial outer membrane and generally consists of three moieties: lipid A, core oligosaccharide (OS), and O-specific polysaccharide or O antigen (OPS) [5]. The core-OS can be subdivided into the inner core and outer core. In *Aeromonas*, the inner core is composed of three α-L,D-heptoses and one phosphorylated α-3-deoxy-D-*manno*-oct-2-ulosomic acid (Kdo). However, the absence of *waaE* in *A. bivalvium*, *A. molluscorum*, *A. rivuli*, *A. fluvialis*, and *A. simiae* suggests two possible inner core LPS models in *Aeromonas*. One, containing the *waaE*, which catalyzes the binding of a β-D-glucose residue to the highly conserved L-α-D-heptose I. The other, containing the *wahX*, which aminoacid sequence shows a β-1-4-glucosyltransferase domain and the function of which has not been described yet. The absence of *waaE* and the presence of an O-antigen ligase domain-containing protein in these species suggest that their outer core-OS may be entirely different from the rest of *Aeromonas* [6]. The latter LPS portion, when present, is the most exposed and variable part of the entire molecule. The OPS is made of oligosaccharide repeating unit (O-units) containing from two to eight different monosaccharide residues (heteropolymers) or, in some bacteria, of identical sugars (homopolymers).

The OPS determines the immunospecificity of different strains and their classification to O-serogroups [7]. This feature is used in epidemiological studies to establish the relationship of bacterial O-serogroups with pathogenicity. The OPS also gives protection to the microorganisms from host defenses, such as complement mediated killing and phagocytosis, and is involved in the interactions of bacteria with plants and bacteriophages [7]. In the case of *Aeromonas* species, 97 O serogroups have been identified up to now, leading to different serological classifications [4]. The finding of O-antigens modifications could be very useful, especially when the identification of *Aeromonas* strains is studied for avoiding infections in aquaculture and for diagnosis of gastrointestinal infections in humans. Despite the large number of *Aeromonas* O-chains characterized [4,8,9,10,11,12], the LPS from *A. bivalvium* has not yet been considered.

In the present study, the structural characterization of the O-specific polysaccharide of the LPS isolated from *Aeromonas bivalvium* strain 868 E^T^ is reported. The structure has been determined by the exploitation of both chemical analysis and NMR spectroscopy.

## 2. Results

### 2.1. LPS Isolation and DOC-PAGE Analysis

**Figure 1:**
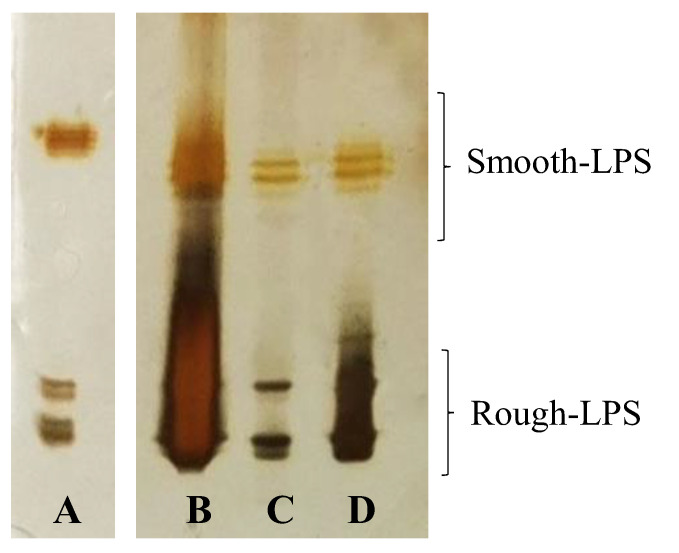
The 14% DOC-PAGE analysis of the extracts after silver staining. Lane (**A**) smooth-LPS of *E. coli* O55:B5 (used as standard); lane (**B**) PCP precipitate; lane (**C**) aqueous extract; (**D**) phenolic phase (see Materials and Methods).

*Aeromonas bivalvium* strain 868 E^T^ (=CECT 7113^T^ = LMG 23376^T^) [13] was grown on tryptic soy broth (TSB) at 30 °C and the LPS was extracted from the cells as reported in the experimental section. The nature of the LPS was established through 14% sodium deoxycholate-polyacrylamide gel electrophoresis (DOC-PAGE) analysis of the extracts after silver staining. A “ladder-like” pattern, typical of smooth-LPS, was clear for the phenol/chloroform/petroleum ether (PCP) precipitate (Figure 1, lane B), and for both aqueous and phenolic phases (Figure 1, lanes C and D, respectively). Moreover, bands at low molecular masses, attributable to LPS molecules lacking the O-chain portion, are also visible for all the extracts (Figure 1).

Since all the extracts contained the same LPS, the aqueous extract was purified as reported in the Experimental section and used for the analysis, due to its higher yields.

### 2.2. Compositional Analysis

The sugar composition of the LPS was achieved by the gas chromatography-mass spectrometry (GC-MS) analysis of the acetylated methyl glycosides (AMGs), that revealed the occurrence of rhamnose (Rha), fucosamine (FucN), galacturonic acid (GalA), glucose (Glc), glucosamine (GlcN), galactosamine (GalN), and a heptose (D,D-Hep). The sugar analysis performed after treatment with 48% aqueous HF (hydrofluoric acid), suggested the additional presence of the Kdo and its phosphorylation (Figure S1).

The fatty acids composition was obtained by GC-MS analysis of their methyl ester derivatives (FAMEs). The fatty acids chromatogram revealed the presence of dodecanoic (C12:0), tetradecanoic (C14:0), and 3-hydroxytetradecanoic [C14:0(3-OH)] acids, as major components, and a small amount of decanoic (C10:0) acid.

### 2.3. Isolation and Chemical Analysis of O-Chain

The LPS was hydrolyzed with 1% acetic acid to cleave the glycosidic linkage between the lipid A and the Kdo of the saccharidic region of the LPS. After centrifugation, the supernatant containing the sugar portion was fractionated by gel filtration chromatography. The sugar analysis performed on the fraction eluted in the void volume and containing the O-chain (named OPS) revealed the presence of rhamnose, fucosamine, glucosamine, and galactosamine (Figure S2). In addition, the retained fractions showed to mainly contain the core oligosaccharides, as revealed by ^1^H-NMR experiments (data not shown). The glycosyl analysis indicated for the core fractions the occurrence of galacturonic acid, glucose and D,D-heptose. Finally, the absolute configuration of all these residues was found to be “D” for GalN, GlcN, Glc, GalA, and FucN, and “L” for Rha.

**Figure 2:**
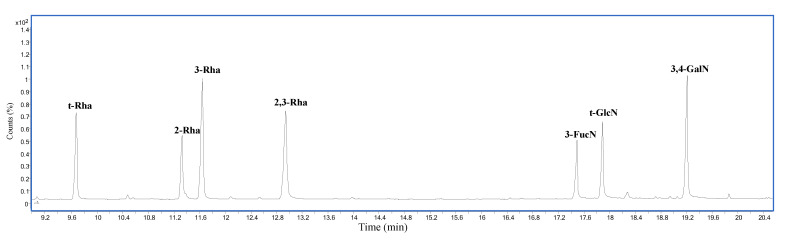
Chromatogram of partially methylated alditol acetates (PMAAs) from the O-chain.

Methylation analysis of the O-chain, performed to establish the monosaccharides linkage position, indicated the presence of a terminal deoxyhexose (t-Rha), 2-substituted deoxyhexose (2-Rha), 3-substituted deoxyhexose (3-Rha), 2,3-disubstituted deoxyhexose (2,3-Rha), 3-substituted-2-amino-2,6-dideoxyhexose (3-FucN), terminal hexosamine (t-GlcN), and 3,4-disubstituted hexosamine (3,4-GalN) (Figure 2).

### 2.4. NMR Analysis of the O-Chain (OPS)

**Figure 3:**
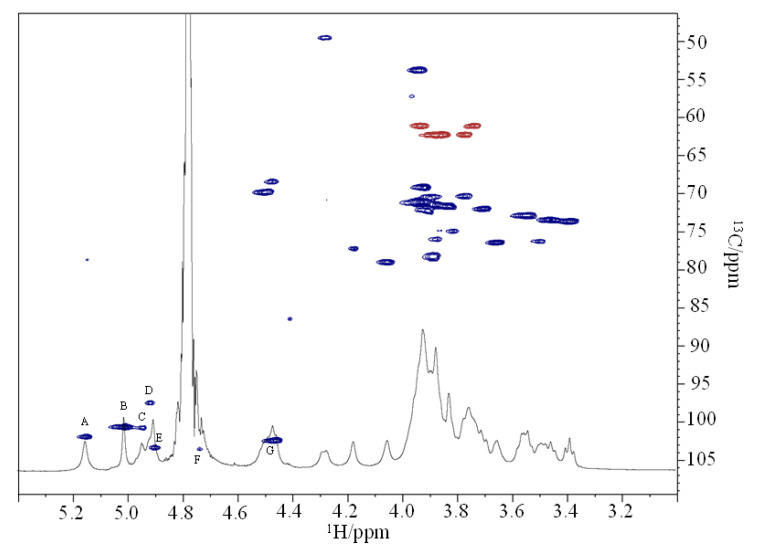
Zoom of ^1^H and ^1^H-^13^C DEPT-HSQC spectra overlapped. The spectra were recorded in D_2_O at 298 K (at 600 MHz).

The repeating unit of the OPS was fully characterized by the following mono- and two-dimensional NMR experiments: double-quantum-filtered phase sensitive correlation spectroscopy (^1^H-^1^H DQF-COSY), total correlation spectroscopy (^1^H-^1^H TOCSY), nuclear Overhauser enhancement spectroscopy (^1^H-^1^H NOESY), heteronuclear single quantum coherence (^1^H-^13^C DEPT-HSQC), and heteronuclear multiple bond correlation (^1^H-^13^C HMBC). Based on the study of NMR spectra (Figure 3 and Figure S3–S7), all the ^1^H and ^13^C chemical shifts of the spin systems were identified (Table 1).

The ^1^H-^13^C DEPT-HSQC experiment (Figure 3) displayed the presence of seven proton signals, between δ 4.5 and 5.4 ppm, that possess cross peaks with anomeric carbons. Spin systems were indicated with a capital letter according to their decreasing chemical shift values (**A**–**G**, Table 1). The anomeric configurations of the residues were assigned by measuring the ^1^*J*_C1,H1_ coupling constants. For **A**, **B**, **C**, **D**, and **E** residues the value of ^1^*J*_C1,H1_ above 170 Hz indicated an α-anomeric configuration, whereas for **F** and **G** residues the value below 165 Hz indicated a β configuration [14].

Residues **A**, **B**, **C**, and **E** were assigned to *manno*-configured residues based on the presence of the correlations in the TOCSY spectrum from H-1 to H-2. These residues were also identified as α-rhamnose, since the TOCSY spectrum showed scalar correlations of the ring protons with methyl signal at δ 1.30, 1.34, 1.30 and 1.30 ppm, respectively. The downfield shift of the C-2 of the residue **A** at δ 79.0 ppm indicated its glycosylation [15]. The residue **B** was identified as a terminal α-rhamnose since none of its carbons were shifted by glycosylation. The spin system **C** was identified as a 2,3-disubstituted rhamnose due to the downfield shift of C-2 and C-3 at δ 77.3 and 78.6 ppm, respectively [15]. Finally, the C-3 downfield shift at δ 76.0 ppm of the residue **E**, compared with an unsubstituted residue indicated glycosylation at this position [15].

The spin systems **D** and **G** were recognized as *galacto*-configured residues, due to the presence of cross-peaks in the TOCSY spectrum from H-1 to H-4. The residue **D** with H-1/C-1 signals at δ 4.92/97.6 ppm was identified as a α-*N*-acetyl galactosamine due to the correlation in the DEPT-HSQC spectrum between its H-2 proton at δ 4.28 ppm and a nitrogen-bearing carbon at δ 49.5 ppm. Moreover, the HMBC spectrum showed correlations between H-2 at δ 4.28 and the carbonyl group at δ 175.3 ppm, which in turn is correlated to the methyl signal at δ 2.04 ppm. The C-3 and C-4 of residue **D** were downfield shifted, respectively, at δ 78.2 and 76.1 ppm with respect to the unsubstituted values, thus evidencing the substitution at these positions [16]. The residue **G** with H-1/C-1 signals at δ 4.46/102.4 ppm was identified as β-*N*-acetyl fucosamine due to the correlation in the DEPT-HSQC experiment of its H-2 proton at δ 3.94 ppm with a nitrogen-bearing carbon at δ 53.7 ppm (Figure 3). Furthermore, both COSY and TOCSY spectra suggested a correlation between the H-5 proton at δ 4.47 ppm and a methyl signal at δ 1.29 ppm. In the HMBC spectrum the H-2 proton correlated with a CO signal at δ 176.0 ppm. The downfield shift of the C-3 carbon resonance at δ 78.5 ppm compared with an unsubstituted residue indicated glycosylation at this position [15].

Finally, **F** was identified as a *gluco*-configured residue since in the TOCSY spectrum starting from H-1 it was possible to assign all the other ring proton resonances. This residue was identified as terminal β-*N*-acetyl glucosamine, based on the correlation of H-1 with H-2 at δ 3.96 ppm in the COSY spectrum and the correlation of H-2 in the DEPT-HSQC experiment with a nitrogen-bearing carbon at δ 57.2 ppm (Figure 3). The HMBC spectrum showed a correlation between H-2 proton at δ 3.96 and the CO group at δ 176.0 ppm, confirming the presence of a *N*-acetyl group. Residue **F** was identified as a terminal GlcN since none of its carbons was shifted by glycosylation [16].

**Figure 4:**
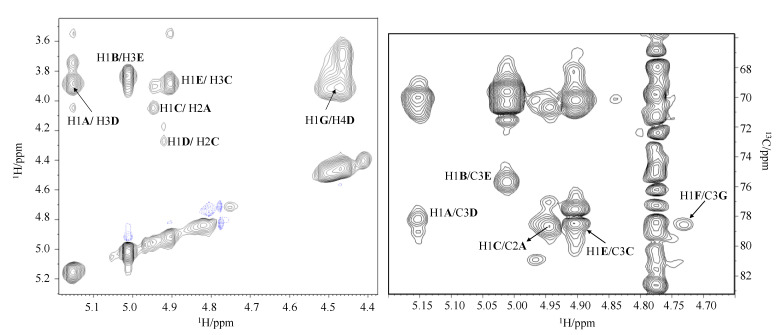
Part of the NOESY (sx) and ^1^H-^13^C HMBC (dx) spectra of O-chain from *A. bivalvium* strain 868 E^T^.

The sequence of the residues was obtained by the analysis of the inter-residual correlations in the NOESY spectrum and from the long-range scalar correlations observed in the ^1^H, ^13^C HMBC spectrum (Figure 4).

The observed inter-residue Nuclear Overhauser Effect (NOE) correlations are H-1 of **A** with H-3 of **D**, H-1 of **B** with H-3 of **E**, H-1 of **D** with H-2 of **C**, H-1 of **C** with H-2 of **A**, H-1 of **E** with H-3 of **C**, and H-1 of **G** with H-4 of **D**. In the anomeric region of the ^1^H, ^13^C HMBC spectrum, the following long-range scalar correlations were observed: H-1 of **A** with C-3 of **D**, H-1 of **B** with C-3 of **E**, H-1 of **C** with C-2 of **A**, H-1 of **E** with C-3 of **C,** and H-1 of **F** with C-3 of **G**. These results agree with the linkage positions obtained from the GC-MS analysis of the PMAAs and the ^13^C glycosylation shifts observed in the DEPT-HSQC experiment. 

**Figure 5:**
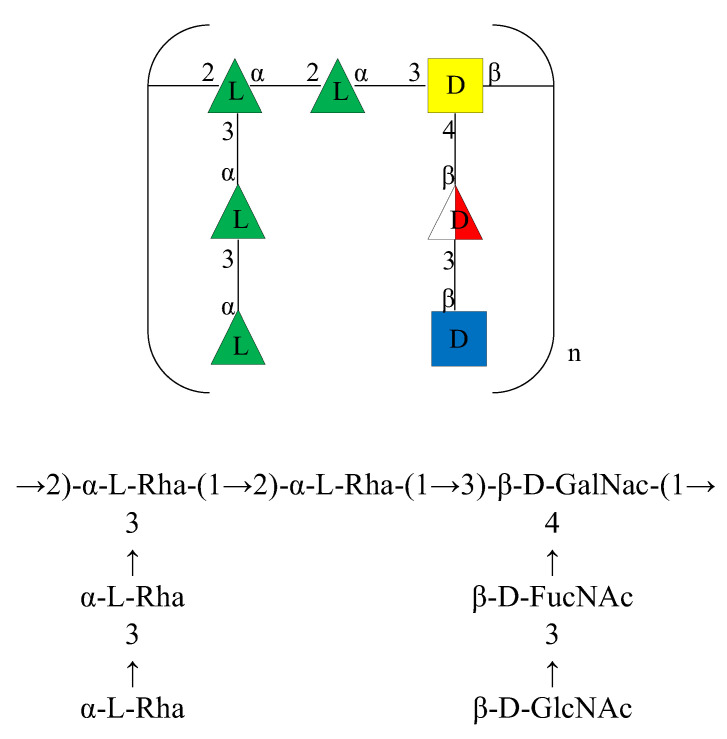
Structure of the O-chain repeating unit from the LPS of *A. bivalvium* strain 868 E^T^ in CFG format (Consortium for Functional Glycomics) (**top**) and standard nomenclature (**bottom**).

Based on the above data, the structure of the OPS of the LPS isolated from *A. bivalvium* strain 868 E^T^ was identified as reported in Figure 5.

It consists of a double branched heptasaccharide containing D-GalNAc, L-Rha, D-GlcNAc, and D-FucNAc residues. Although these residues have already been found in other O-chains from *Aeromonas* genus [17,18,19], the structure of the repeating unit was found to be uncommon among the O-chains characterized of species belonging to this genus as well as in other bacterial polysaccharide structures.

## 3. Materials and Methods

### 3.1. Bacterial Characteristics, Isolation and Growth

*A. bivalvium* does not cluster within any of the 20 *Aeromonas* DNA hybridization groups and *Aeromonas* genomospecies described [13]. Three or more biochemical tests allow differentiation of *A. bivalvium* from all the recognized *Aeromonas* species except *A. caviae* and *A. media*. A positive reaction in the lysine decarboxylase test allows separation from *A. caviae* and *A. media* [7]. In addition, production of ONPG, brown pigment, fermentation of lactose and mannose and utilization of lactose, mannose, and raffinose are also differential tests to *A. media*. A positive reaction in the utilization of glycerol and acid production from it also allows separation to *A. caviae* [13].

*Aeromonas bivalvium* strain 868 E^T^ (=CECT 7113^T^ = LMG 23376^T^) [9] was isolated from cockles (*Cardium* sp.) obtained from a retail market in Barcelona (Spain) in 2007 [13], has a DNA G + C content of 62.6 mol% and its GenBank/EMBL/DDBJ accession numbers for the 16S rRNA gene sequence is DQ504429.

This strain was grown on tryptic soy broth (TSB, Conda-Pronadisa, Candalab, Madrid, Spain) or tryptic soy agar (TSA, Conda-Pronadisa, Candalab, Madrid, Spain) at 30 °C overnight. Five-liter growths were washed with water and dehydrated by sequential washing with methanol:chloroform (1:1, three times; Panreac, Castellar del Vallès, Spain), ethanol (Panreac, Castellar del Vallès, Spain), acetone (two times; Panreac, Castellar del Vallès, Spain), and diethyl ether (Merck-Life Science, Mollet del Vallès, Spain). Dried cells were obtained after evaporation of the solvents.

### 3.2. LPS Isolation

Dried cells (5.5 g) were extracted by using the PCP (phenol/chloroform/petroleum ether 40–60 °C, 2:5:8 *v/v/v*) method [20]. All the solvents used for the extraction were purchased from Merck-Life Science (Rome, Italy).

After removal of chloroform and petroleum ether at reduced pressure, the phenol residue was treated with water to precipitate the LPS. The precipitate was removed and freeze-dried (23.4 mg, yield 0.4%) and the phenolic phase was dialyzed against water (cut-off 3500 Da, Spectra/Por, VWR Chemicals, Milan, Italy) and freeze dried (11.6 mg, yield 0.6%). The cells debris were then extracted with the hot phenol water method [21].

The obtained aqueous phase was purified from proteins and nucleic acids by treatment with DNase from bovine pancreas, RNase from bovine pancreas at 37 °C for 16 h and then with Proteinase K from *Tritirachium album* at 60 °C for 2 h (Merck-Life Science, Rome, Italy). The mixture was dialyzed against water (cut-off 3500 Da, Spectra/Por, VWR Chemicals, Milan, Italy) and freeze-dried (469 mg). The yield of the LPS from the aqueous phase was 8.5%. All the yields have been calculated as reported: (mg of extract/mg of cells) × 100.

### 3.3. DOC-PAGE Analysis

All the extracts were screened by 14% PAGE with sodium deoxycholate (DOC) as the detergent by using the Laemmli’s system [22] and stained in accordance with Tsai’s procedure [23].

### 3.4. Chemical Analyses

Monosaccharides were analyzed by GC-MS as acetylated methyl glycosides (AMGs) derivatives. Firstly, 0.5 mg of the aqueous extract was treated with 100 μL of HF (48% aq.; CARLO ERBA Reagents, Cornaredo (MI), Italy); then the methanolysis reaction was performed. Briefly, HCl/CH_3_OH (1 mL, 1.25 M; Merck-Life Science, Rome, Italy) was added to the sample, and the reaction was performed at 80 °C for 16 h [24]. The resulting mixture was extracted three times with hexane (VWR Chemicals, Milan, Italy). The hexane layer, containing the fatty acids as methyl esters, was directly analyzed by GC-MS. The methanol layer, containing the methyl glycosides was dried, and acetylated with Ac_2_O and pyridine (50 µL, 100 °C, 30 min, Merck-Life Science).

The absolute configuration of the sugars was determined by gas-chromatography analysis of their acetylated (*R*)-2-octyl glycosides derivatives [25].

The linkage positions of the monosaccharides were obtained by the analysis of the partially methylated alditol acetates (PMAAs). The methylation reaction was achieved by incubating 2 mg of the LOS sample with CH_3_I (100 µL; Merck-Life Science, Rome, Italy) and NaOH powder in dimethyl sulfoxide (DMSO, 300 µL; Merck-Life Science, Rome, Italy) for 20 h [26]. The product was then hydrolyzed with 2 M trifluoroacetic acid (120 °C, 2 h; Merck-Life Science, Rome, Italy), reduced with NaBD_4_ (Merck-Life Science, Rome, Italy), and acetylated.

All the samples were analyzed on an Agilent Technologies gas chromatograph 7820A equipped with a mass selective detector 5977B and an HP-5 capillary column (Agilent, Milan, Italy 30 m × 0.25 mm i.d., flow rate 1 mL/min, He as carrier gas). MGA were analyzed using the following temperature program: 140 °C for 3 min, then 140→240 °C at 3 °C/min. The temperature program for octyl glycosides was performed at 150 °C for 5 min, then 150 °C→240 °C at 6 °C/min, and 240 °C for 5 min. The temperature program for PMAAs is the following: 90 °C for 1 min, then 90→140 °C at 25 °C/min, then 140→200 °C at 5 °C/min, then 200→280 °C at 10 °C/min, and finally 280 °C for 10 min. Finally, the following temperature program was used for the fatty acids analysis: 110 °C for 3 min, 140 to 280 °C at 10 °C/min.

### 3.5. Isolation of O-Chain

The LPS was hydrolyzed with 1% aqueous CH_3_COOH (100 °C, 3 h; Merck Life Science, Rome, Italy) [27]. The obtained suspension was centrifuged (8000 rpm, 4 °C, 30 min). The precipitate (lipid A) was washed twice with water and lyophilized. The supernatant, containing the saccharide portion, was fractioned on a Biogel P-10 column (Bio-Rad, Segrate (MI), Italy 0.75 cm × 95 cm, flow rate 11.4 mL/h, fraction volume 2.5 mL) and eluted with water. The obtained fractions were then lyophilized [28].

### 3.6. NMR Spectroscopy

All NMR spectra were recorded using a Bruker 600 MHz spectrometer (Bruker, Milan, Italy) equipped with a cryoprobe. All two- dimensional homo- and heteronuclear experiments (^1^H-^1^H COSY, ^1^H-^1^H TOCSY, ^1^H-^1^H NOESY, ^1^H-^13^C DEPT-HSQC, ^1^H-^13^C HSQC-TOCSY, and ^1^H-^13^C HMBC) were performed using standard pulse sequences available in the Bruker software. NMR spectra were recorded at 298 K, and the mixing time for the TOCSY and NOESY experiments was 100 ms [29]. Chemical shifts were measured in D_2_O (99.9% atom D, Merck-Life Science, Rome, Italy) using acetone (Merck-Life Science, Rome, Italy) as internal standard (δ_H_ 2.225 and δ_C_ 31.45 for proton and carbon, respectively).

## 4. Conclusions

In this paper we reported the isolation of the LPS and the structural characterization of the O-chain from *Aeromonas bivalvium* strain 868 E^T^.

The OPS structure was obtained by means of chemical analyses and NMR spectroscopy, revealing a branched heptasaccharide repeating unit. The detailed structural characterization of O-antigens coming from *Aeromonas* species still plays a pivotal role in extending the relationship between the LPS structure and the pathogenicity of this water-born genus, which could help to diminish the risk of public health.

Finally, the presence of the galacturonic acid and of only the D,D-heptose isomer in the core region confirmed the genome analysis, suggesting an uncommon core region respect to other *Aeromonas* strains.

## Figures and Tables

**Table 1:** ^1^H and ^13^C NMR chemical shifts (δ, ppm) of the O-Chain from *A. bivalvium* strain 868 E^T^.

Residue	H1 C1 ^1^*J*_C1,H1_	H2 C2	H3 C3	H4 C4	H5 C5	H6 C6	CH_3_CO	CO
α-2-L-Rha **A**	5.15 102.0 181 Hz	4.05 79.0	3.92 71.1	3.46 73.4	3.77 70.3	1.30 17.9		
α-t-L-Rha **B**	5.01 100.6 179 Hz	3.83 71.6	3.91 70.4	3.39 73.6	4.49 69.8	1.34 18.0		
α-2,3-L-Rha **C**	4.94 100.7 178 Hz	4.17 77.3	3.95 78.6	3.54 72.8	3.89 70.4	1.30 17.9		
α-3,4-D-GalNAc **D**	4.92 97.6 180 Hz	4.28 49.5	3.88 78.2	3.66 76.1	3.91 72.2	3.77, 3.86 62.2	2.04 23.4	175.3
α-3-L-Rha **E**	4.90 103.3 179 Hz	3.87 71.5	3.87 76.0	3.54 70.7	3.74 70.5	1.30 17.8		
β-t-D-GlcNAc **F**	4.74 103.6 164 Hz	3.96 57.2	3.87 74.8	3.81 71.6	3.50 76.2	3.74, 3.93 61.1	2.04 23.4	176.0
β-3-D-FucNAc **G**	4.46 102.4 163 Hz	3.94 53.7	3.92 78.5	3.70 71.9	4.47 68.5	1.29 16.8	2.04 23.4	176.0

## Data Availability

The data presented in this study are contained within the article.

## Supplementary Materials

The following supporting information can be downloaded at: https://www.mdpi.com/article/10.3390/ijms23031204/s1.
