# Supplementary material for: Complete Characterization of the O-Antigen from the LPS of Aeromonas bivalvium
Source: Int J Mol Sci. 2022 Jan 21;23(3):1204. doi: 10.3390/ijms23031204 (PMC8835325; doi:10.3390/ijms23031204)
Supplement: Supplementary file 1 [file ijms-23-01204-s001.zip › ijms-1542358-supplementary.pdf]

Supporting Information

Complete characterization of the O-antigen from the LPS of *Aeromonas bivalvium*

Rossella Di Guida[1], Angela Casillo[1], Juan M. Tomás[2], Susana Merino[2] and Maria Michela Corsaro[1*]

[1] Department of Chemical Sciences, University of Naples "Federico II", Complesso Universitario Monte S. Angelo, Via Cintia 4, 80126 Naples, Italy; ross.diguida@gmail.com; angela.casillo@unina.it; corsaro@unina.it

[2] Departamento de Genética, Microbiología y Estadística, Sección Microbiología, Virología y Biotecnología, Facultad de Biología, Universidad de Barcelona, Avd. Diagonal 643, 08028 Barcelona, Spain; jtomas@ub.edu; smerino@ub.edu

* Correspondence: corsaro@unina.it

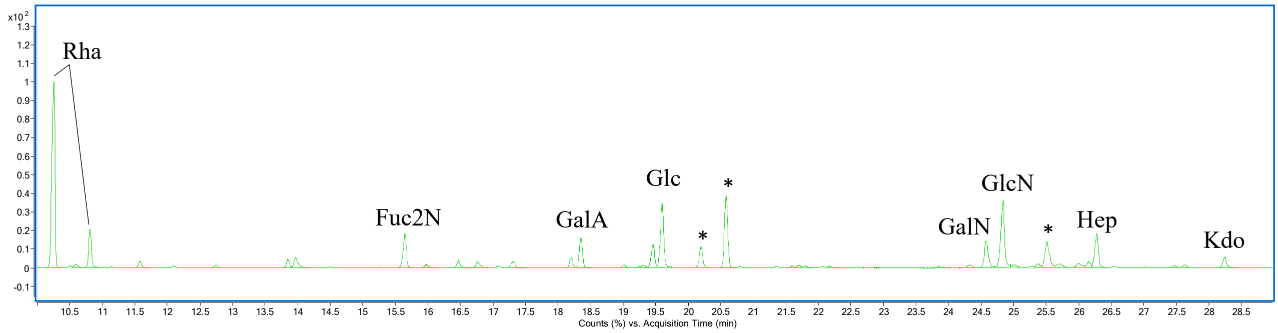

Figure S1. GC-MS chromatogram of AMG of the LPS from *A. bivalvium* strain 868E^T. The peaks marked with asterisk are contaminants.

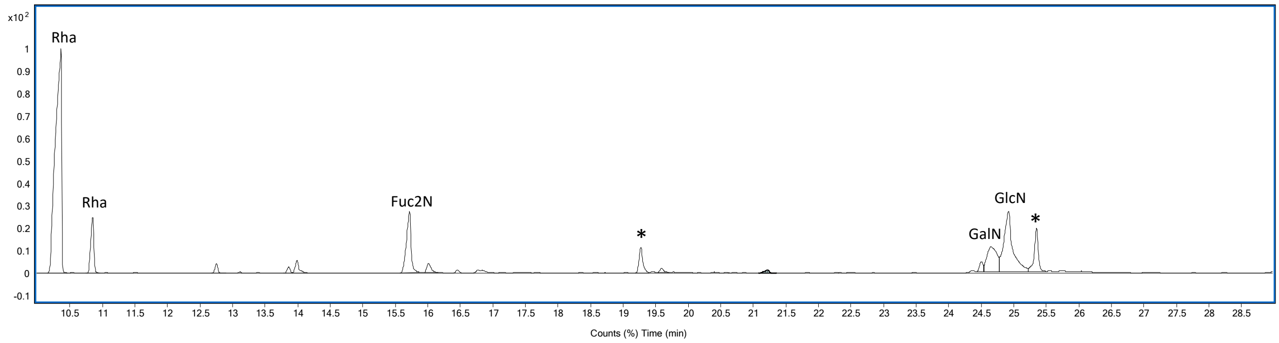

Figure S2. chromatogram of AMG of the purified OPS from *A. bivalvium* strain 868E^T. The peaks marked with asterisk are contaminants.

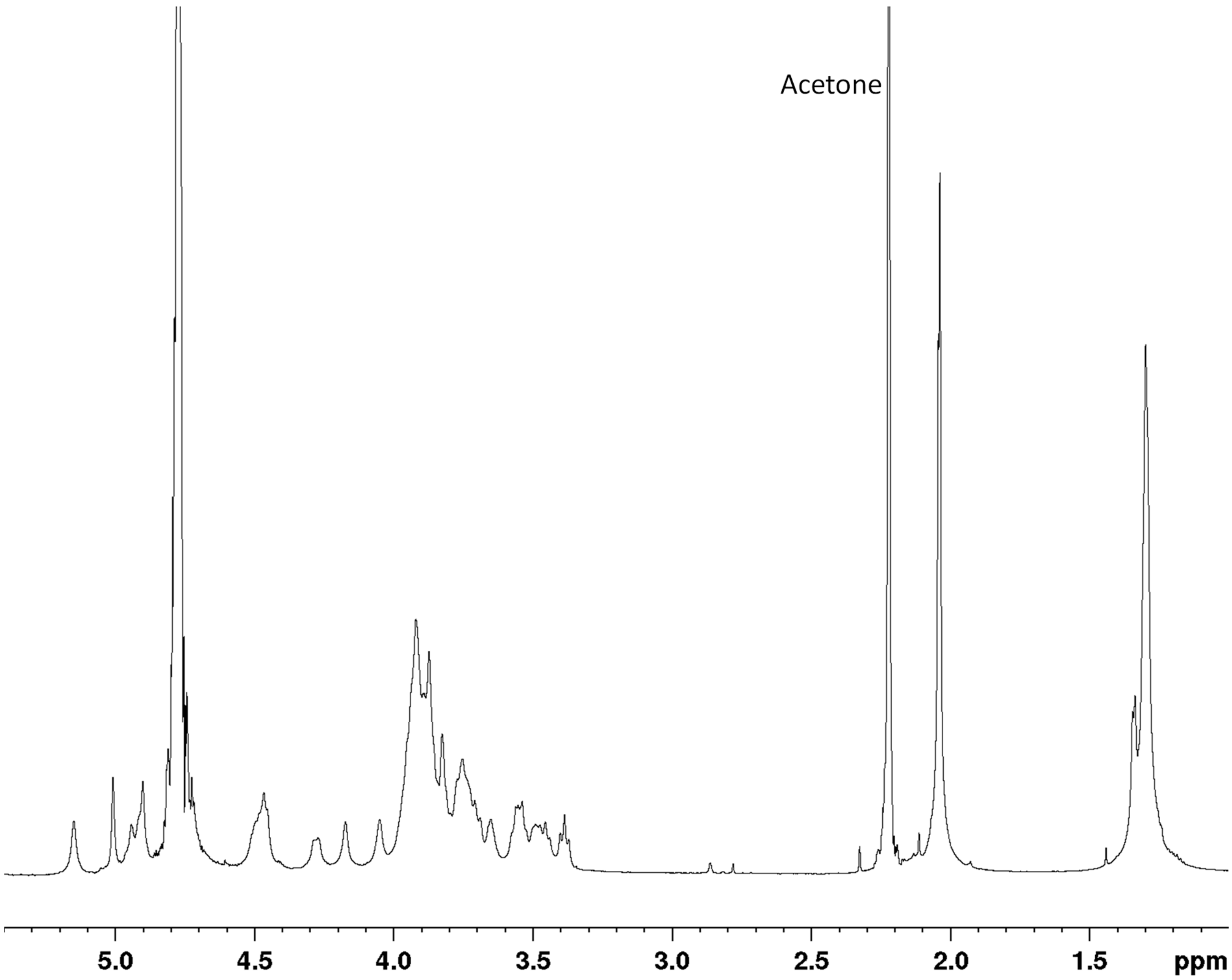

Figure S3. ^{1}H NMR spectrum of the OPS from *A. bivalvium* strain 868E^T. The spectrum was recorded in D_2O at 298K.

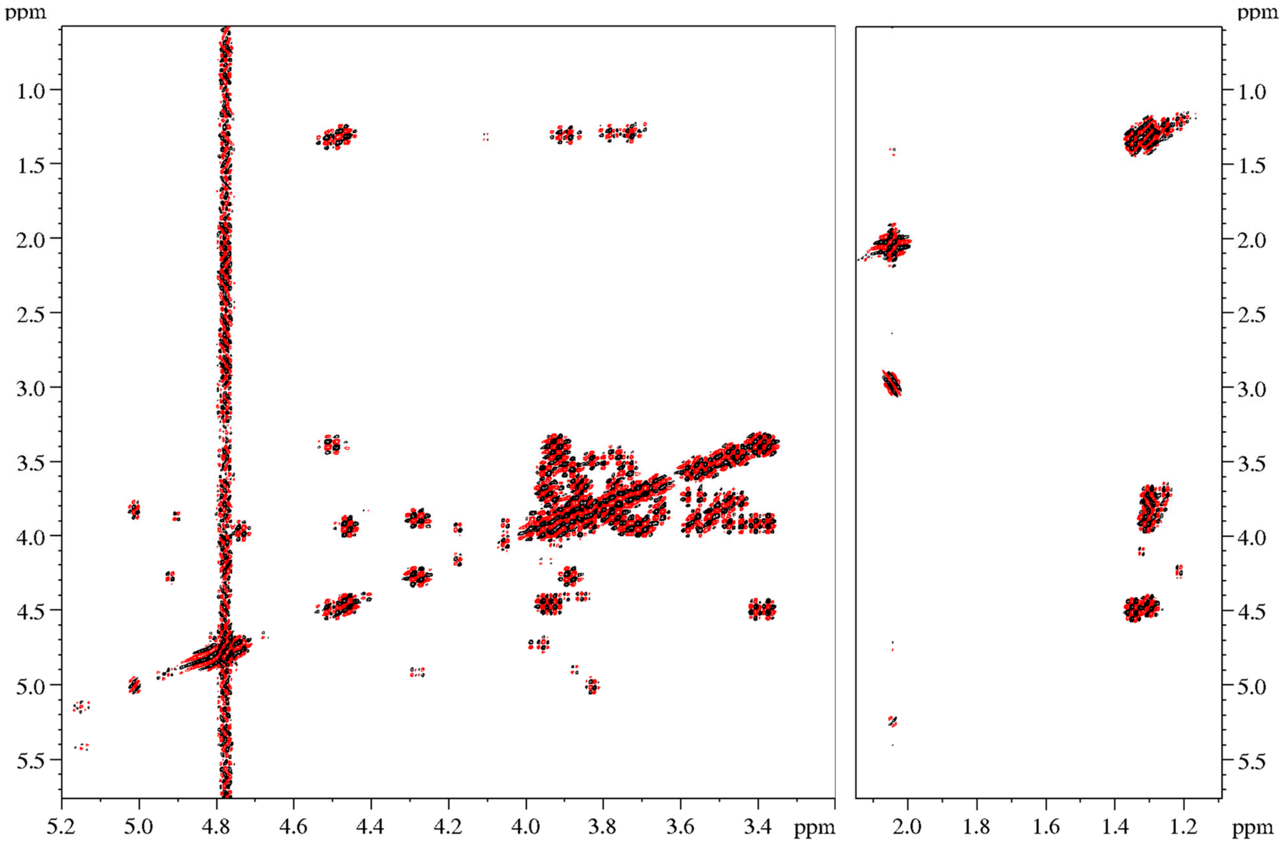

Figure S4. Selected regions of ^{1}H-^{1}H COSY spectrum of the OPS from *A. bivalvium* strain 868E^{T}. The spectrum was recorded in D_2O at 298K.

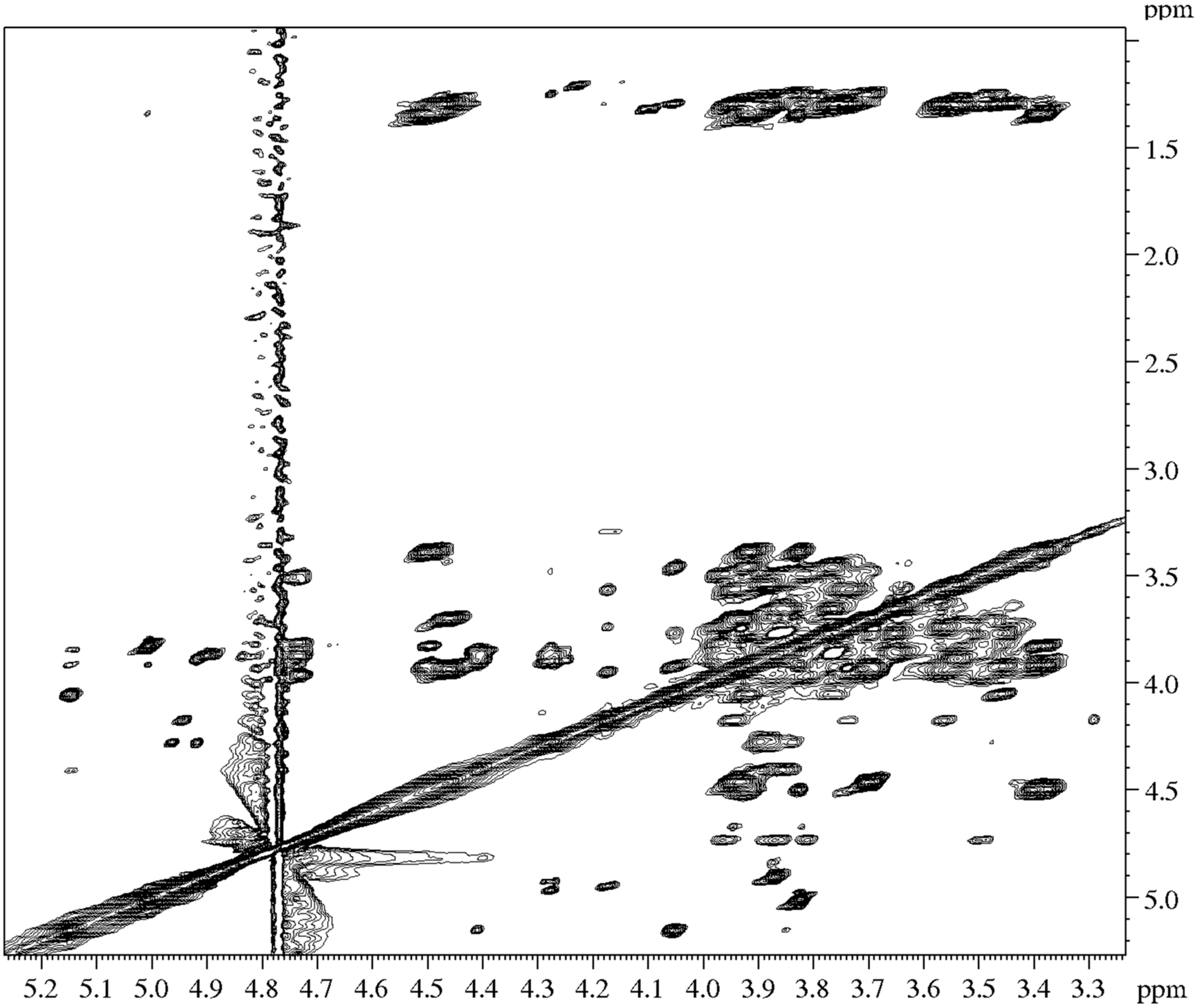

Figure S5. Selected region of ^{1}H-^{1}H TOCSY spectrum of the OPS from *A. bivalvium* strain 868E^{T}. The spectrum was recorded in D_2O at 298K.

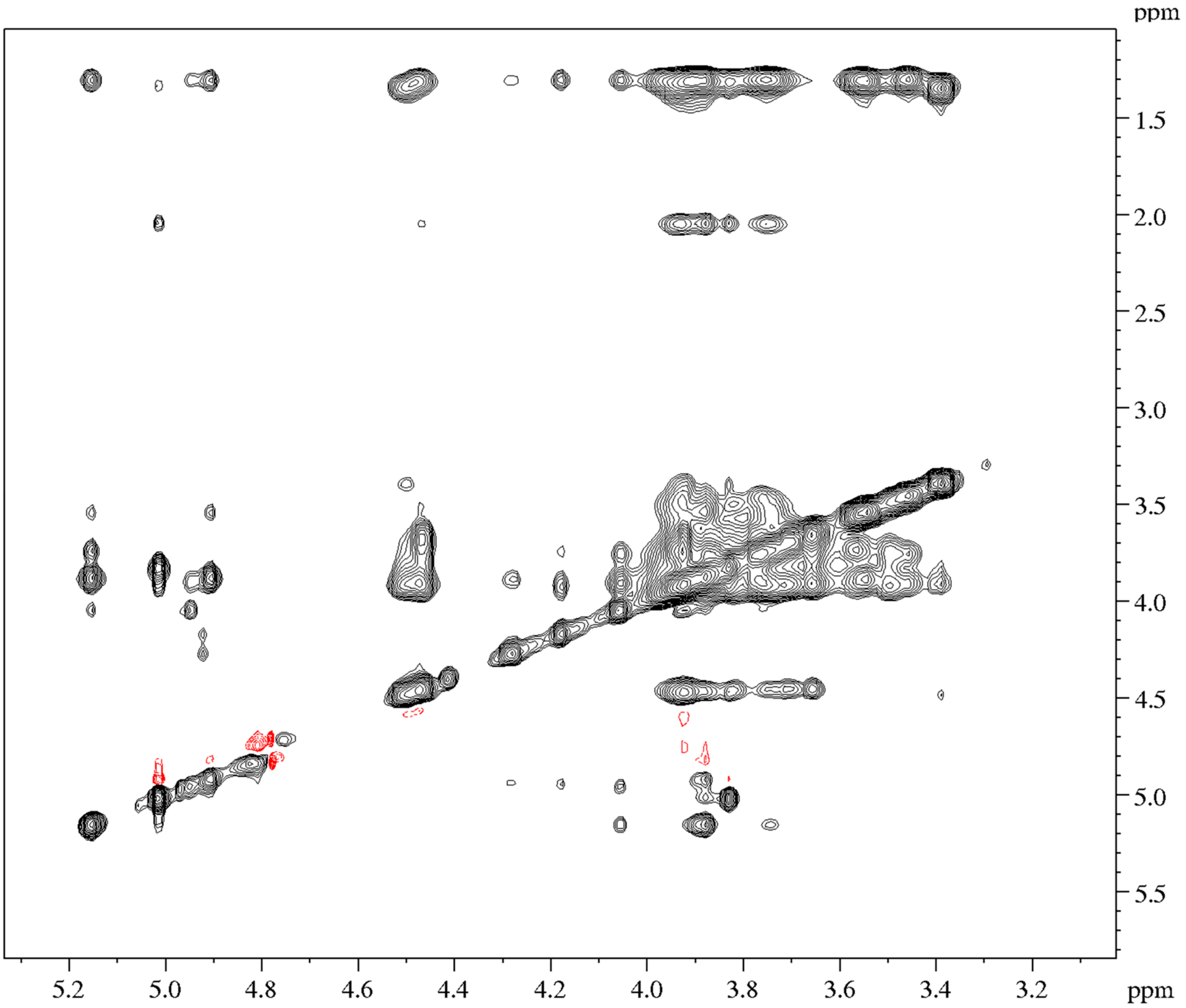

Figure S6. Selected region of ^1H-^1H NOESY spectrum of the OPS from *A. bivalvium* strain 868E^T. The spectrum was recorded in D_2O at 298K.

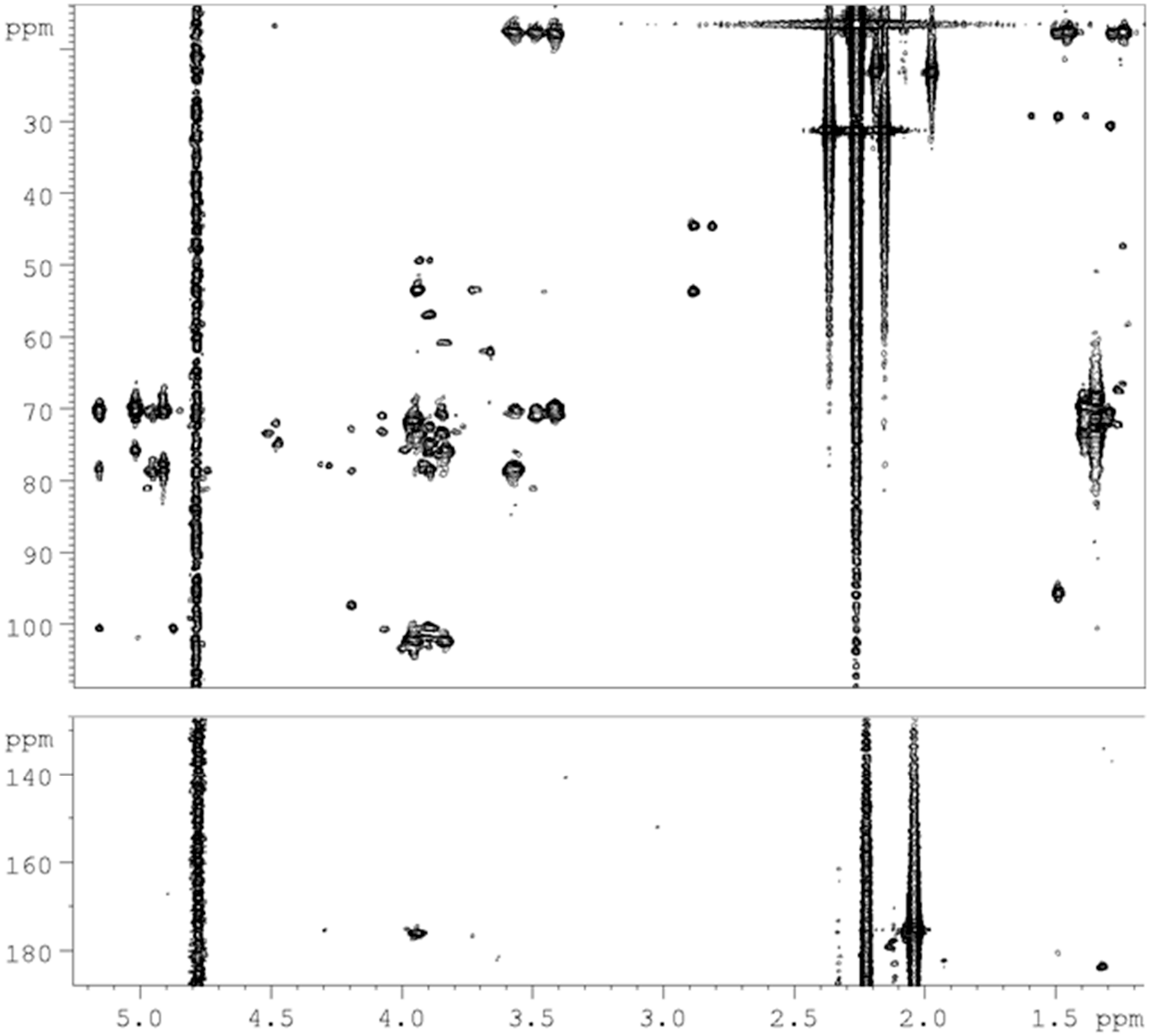

Figure S7. ^{1}H-^{13}C HMBC spectrum of the OPS from *A. bivalvium* strain 868E^{T}. The spectrum was recorded in D_2O at 298K.
